# Stanford neuromodulation therapy for treatment-resistant depression: a systematic review

**DOI:** 10.3389/fpsyt.2023.1290364

**Published:** 2023-12-13

**Authors:** Xian-Jun Lan, Dong-Bin Cai, Qi-Man Liu, Zhen-Juan Qin, Saxby Pridmore, Wei Zheng, Yu-Tao Xiang

**Affiliations:** ^1^The Brain Hospital of Guangxi Zhuang Autonomous Region, Liuzhou, China; ^2^Shenzhen Traditional Chinese Medicine Hospital, Shenzhen, China; ^3^The Affiliated Brain Hospital of Guangzhou Medical University, Guangzhou, China; ^4^Discipline of Psychiatry, University of Tasmania, Hobart, TAS, Australia; ^5^Unit of Psychiatry, Department of Public Health and Medicinal Administration, Institute of Translational Medicine, Faculty of Health Sciences, University of Macau, Macau, Macao SAR, China; ^6^Centre for Cognitive and Brain Sciences, University of Macau, Macau, Macao SAR, China

**Keywords:** stanford neuromodulation therapy, treatment-resistant depression, response, remission, systematic review

## Abstract

**Objective:**

This systematic review of randomized controlled studies (RCTs) and observational studies evaluated the efficacy and safety of stanford neuromodulation therapy (SNT) for patients with treatment-resistant depression (TRD).

**Methods:**

A systematic search (up to 25 September, 2023) of RCTs and single-arm prospective studies was conducted.

**Results:**

One RCT (*n* = 29) and three single-arm prospective studies (*n* = 34) met the study entry criteria. In the RCT, compared to sham, active SNT was significantly associated with higher rates of antidepressant response (71.4% versus 13.3%) and remission (57.1% versus 0%). Two out of the three single-arm prospective studies reported the percentage of antidepressant response after completing SNT, ranging from 83.3% (5/6) to 90.5% (19/21). In the three single-arm prospective studies, the antidepressant remission rates ranged from 66.7% (4/6) to 90.5% (19/21). No severe adverse events occurred in all the four studies.

**Conclusion:**

This systematic review found SNT significantly improved depressive symptoms in patients with TRD within 5 days, without severe adverse events.

## Introduction

Major depressive disorder (MDD) is a leading cause of disability worldwide (1), and up to 55% of patients suffering from MDD fulfill the criteria of treatment-resistant depression (TRD) (2). Accumulating evidence has found that ketamine (3) and esketamine (4) had a rapid antidepressant, antisuicidal effects on TRD. Esketamine nasal spray has been approved as the first therapeutic agent for TRD (5). Furthermore, a real-world study found a significant reduction of depressive symptoms in patients suffering from TRD after receiving esketamine nasal spray (5). Apart from antidepressant medication, strategies such as vagus nerve stimulation (6), electroconvulsive therapy (7, 8), transcranial alternating current stimulation (9), and transcranial magnetic stimulation (TMS) [e.g., deep TMS (10), accelerated TMS (11), intermittent theta-burst stimulation (iTBS) (12), accelerated iTBS (13), bilateral TBS (14), and continuation TBS (15)], have been developed as a nonpharmacological alternative for the treatment of MDD.

iTBS has been approved in many countries in the treatment of TRD. However, efficiency has been less than desired and another treatment protocol (number and spacing of individual treatments) may provide a better outcome (16). Stanford neuromodulation therapy (SNT), a neuroscience-informed accelerated iTBS protocol, had been investigated as a solution to these limitations (17). For example, Cole et al. reported significant superiority of active SNT over sham stimulation in improving depressive symptoms in TRD (17). We conducted this systematic review of randomized controlled studies (RCTs) and single-arm prospective studies to examine the efficacy and safety of SNT for patients with TRD.

## Method

### Inclusion criteria

Following PICOS acronym, studies were selected and screened by three investigators (XJL, ZJQ and QML) for inclusion in this systematic review according to the Preferred Reporting Items for Systematic Reviews and Meta-analyses (PRISMA) reporting guideline (18). Participants: patients with TRD based on study-defined diagnostic criteria. For example, TRD was defined as failure to responding to at least two antidepressants from different classes at adequate dosages (19). Intervention vs. Comparison: active SNT plus antidepressants or antidepressants free versus sham SNT plus antidepressants or antidepressants free in RCTs; or SNT added to antidepressants or antidepressants free in single-arm prospective studies. Outcomes: Coprimary outcomes were study-defined response and remission. A secondary outcome was adverse events. Study: only published RCTs or single-arm prospective studies on the efficacy and safety of SNT, using resting-state functional connectivity Magnetic Resonance Imaging (fcMRI) to target high-dose iTBS (10 sessions of iTBS daily, 18,000 pulses/day, 5 consecutive days, and 90,000 total pulses), as an adjunctive treatment for TRD were considered. High-dose iTBS studies with different intervals between sessions, such as 50-min or 60-min, were approved. Studies on patients without TRD were excluded (20). Systematic reviews, retrospective studies, and case reports/series were not included.

### Study selection

We performed a systematic review of relevant literature from inception to 25 September, 2023, based on the Cochrane Library, PubMed, EMBASE and PsycINFO databases and reference lists from retrieved studies (16, 17, 21) to identify RCTs and single-arm prospective studies (single-group and before-after design) that examined the antidepressant effects of SNT for TRD. The following search terms were used: (“Stanford neuromodulation therapy” OR “Stanford accelerated intelligent neuromodulation therapy” OR SNT OR “High-dose spaced theta-burst stimulation”) AND (depress* OR dysphor* OR dysthymi* OR melanchol* OR antidepress* OR bipolar OR MDD). Study selection was performed independently by three investigators (XJL, ZJQ and QML).

### Data extraction

Data extraction was performed independently by three investigators (XJL, ZJQ, and QML). If there were discrepancies, consensus was achieved between the investigators and then discussion was conducted with a senior investigator (WZ). Additionally, the first and/or corresponding authors were contacted as necessary to acquire any pertinent information that was missing.

### Quality assessment

For RCTs and single-arm prospective studies, the Cochrane risk of bias (22) and Risk Of Bias In Non-randomized Studies – of Interventions (ROBINS-I) (23) were, respectively, used to assess the study quality independently by the three investigators (XJL, ZJQ, and QML).

## Results

**Figure 1:**
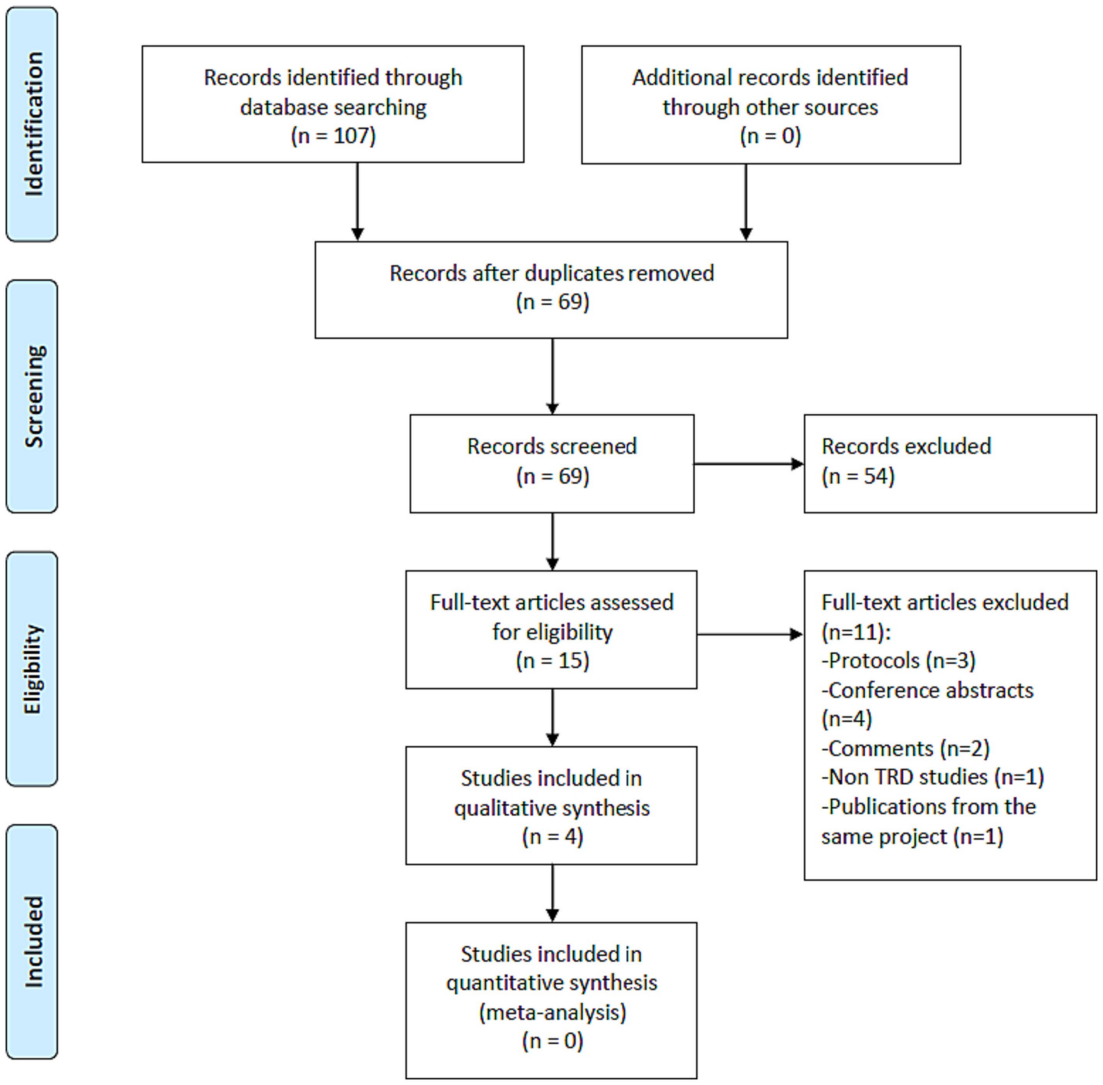
PRISMA flow diagram. PRISMA, Preferred Reporting Items for Systematic Reviews and Meta-analyses; RCTs, randomized controlled trials; TRD, treatment-resistant depression.

As shown in Figure 1, 107 potentially relevant articles were identified, and finally one RCT (17) and three single-arm prospective studies (16, 21, 24) met the study entry criteria (Table 1). Four studies (*n* = 63) (16, 17, 21, 24) examined the efficacy and safety of adjunctive SNT for adult patients with TRD. The risk of bias of included studies is summarized in Tables 2, 3. Based on the Cochrane risk of bias tool, the double-blind RCT (17) was rated as low risk with regard to attrition bias and reporting bias (Table 2). In the RCT, compared to sham, active SNT was significantly associated with higher rates of antidepressant response (71.4% versus 13.3%) and remission (57.1% versus 0%) (17). Two out of the three single-arm prospective studies reported the rates of antidepressant response after completing SNT, ranging from 83.3% (5/6) (21) to 90.5% (19/21) (16). In the three single-arm prospective studies, the antidepressant remission rates ranged from 66.7% (4/6) (21), 83.3% (5/6) (24) to 90.5% (19/21) (16). Furthermore, Cole et al. found 70% of patients with TRD continued to fulfill response criteria at 1-month follow-up (16). Poydasheva et al. reported that 40% of patients with TRD met the criteria for both response and remission at the 3-month follow-up assessment (24). No severe adverse events occurred in the four studies (16, 17, 21).

**Table 1:** Summary of studies included in this systematic review.

Study (country)	Sample size (n)^a^	Design: -Blinding -Setting (%) -Treatment duration (days)	Participants: -Diagnosis (%) -Diagnostic criteria -Illness duration^c^ (yrs)	-Mean age^c^ (yrs) (range) -Sex: male (%)	-TRD criteria -Clinical effects	SNT therapeutic frequency and ADs dosages (mg/day); Number of patients (n)	-Stimulation target (active/sham)^b^ -Intensity (%rMT)	-Pulses/day (total pulses) -Intersession interval per session -Number of sessions (n/day)	Depressive symptoms measured by MADRS or HRSD (Pre/Post-SNT and follow-up at any time)	Response and remission rate (Post-SNT and follow-up at any time)
Cole et al., 2020 (USA)	22	-Observational study -Outpatients −5	-MDD (90.5) and BD (9.5) -DSM-5 −23.0	−44.9 (19–78) −9 (42.9)	- ≥ 1 ADs -MADRS	Active SNT (50 Hz) + ADs (NR); *n* = 21^c^	-Left DLPFC −90	−18,000 (18,000*5 days = 90,000) −50 min −50 (10/day)	Pre-SNT: 34.86 ± 5.29 Post-SNT: 5.0 ± 6.37; 1-month follow-up: 10.95 ± 11.76	90.5 and 90.5% (Post-SNT); 70 and 60% (1-month follow-up)
Cole et al., 2022 (USA)	29	-DB -NR −5	-MDD (100) -DSM-5 −23.4	−50.6 (22–80) −19 (65.5)	-NR -MADRS	1. Active SNT (50 Hz) + ADs (NR) or ADs free; *n* = 14 2. Sham SNT (no active stimulation) + ADs (NR) or ADs free; *n* = 15	-Left DLPFC −90	−18,000 (18,000*5 days = 90,000) −50 min −50 (10/day)	Pre-SNT: 31.0 ± 4.0 Post-SNT: NR Pre-sham: 35.0 ± 6.0 Post-sham: NR	Active SNT: 71.4 and 57.1% (Post-SNT); 77.8 and 66.7% (1-week follow-up); 84.6 and 53.8% (2-week follow-up); 69.2 and 61.5% (3-week follow-up); 69.2 and 46.2% (4-week follow-up) Sham SNT: 13.3 and 0% (Post-sham); 20.0 and 10.0% (1-week follow-up); 7.1 and 7.1% (2-week follow-up); 7.1 and 7.1% (3-week follow-up); 7.1 and 0% (4-week follow-up)
Poydasheva et al., 2022 (Russia)	6	-Observational study -NR −5	-MDD (33.3) and BD (66.7) -ICD-10 −21.2	−40.2 (21–66) −3 (50)	-NR -MADRS	Active SNT (50 Hz) + ADs (NR); *n* = 6	-Left DLPFC −120	−18,000 (18,000*5 days = 90,000) −1 h −50 (10/day)	Pre-SNT: 19.83 ± NR Post-SNT: NR	NR and 83.3% (Post-SNT); NR and 20% (1-month follow-up)^d^; 80 and 60% (2-month follow-up)^d^; 40 and 40% (3-month follow-up)^d^
Williams et al., 2018 (USA)	6	-Observational study -NR −5	-MDD (83.3) and BD (16.7) -DSM-5 −32.0	−56.0 (38–69) −2 (33.3)	-NR -HRSD	Active SNT (50 Hz) + ADs (NR); *n* = 6	-Left DLPFC −90	−18,000 (18,000*5 days = 90,000) −50 min −50 (10/day)	Pre-SNT: 28.8 ± 6.0 Post-SNT: 7.0 ± 4.7	83.3 and 66.7% (Post-SNT); 33.3 and 0% (2-week follow-up); 0 and 0% (4-week follow-up)

^a^Overall number of participants.

^b^The left DLPFC functional target was localized for each participant using the Localite Neuronavigation System.

^c^It was extracted from the available data of each study.

^d^The follow-up data was analyzed from a cohort of five patients, as one patient withdrew from the study after the stimulation completion.

ADs, antidepressants; APs, Antipsychotics; BD, bipolar disorder; DB, double blind; DSM-5, Diagnostic and Statistical Manual of Mental Disorders 5th edition; DLPFC, dorsolateral prefrontal cortex; HRSD, Hamilton Rating Scale for Depression; h, hour; ICD-10, International Classification of Diseases, 10th edition; MADRS, Montgomery-Asberg Depression Rating Scale; MDD, major depressive disorder; min, minutes; NR, not reported; rMT, resting motor threshold; SNT, Stanford Neuromodulation Therapy; TRD, treatment-resistant depression; yrs, years.

**Table 2:** Cochrane risk of bias.

	*Random sequence generation (selection bias)*	*Allocation concealment (selection bias)*	*Blinding of participants and personnel*	*Blinding of outcome assessment (Symptom reduction, response)*	*Incomplete outcome data addressed (attrition bias)*	*Selective reporting (reporting bias)*	*Other sources of bias*
Cole et al., 2022 (USA)	?	?	+	++	+	+	?

+, Low risk of bias; −, High risk of bias; ?, Unclear risk of bias; nd, not determined.

**Table 3:** Risk of bias in single-arm prospective studies of SNT for TRD with ROBINS-I tool.

Study (country)	Bias due to confounding	Bias in selection of patients into the study	Bias in classification of intervention	Bias due to deviations from intended interventions	Bias due to missing data	Bias in measurement of outcomes	Bias in selection of the reported result	Overall risk
Cole et al., 2020 (USA)	Moderate	Moderate	Low	Low	Low	Low	Low	Moderate
Poydasheva et al., 2022 (Russia)	Moderate	Moderate	Low	Low	Low	Low	Low	Moderate
Williams et al., 2018 (USA)	Moderate	Moderate	Low	Low	Low	Low	Low	Moderate

Notes: A study was assigned moderate risk if the study was judged to be at low or moderate risk for all domains. A study was assigned critical risk if 1 or more of domains was rated as critical risk.

ROBINS-I, Risk Of Bias In Non-randomized Studies – of Interventions; SNT, Stanford neuromodulation therapy; TRD, treatment-refractory depression.

## Discussion

This systematic review found SNT, using resting-state fcMRI to target high-dose iTBS, could significantly improve depressive symptoms in patients with TRD within 5 days, without severe adverse events. The rate of antidepressant remission (66.7–90.5%) reported in the included studies is higher than the corresponding figures for ketamine treatment (8.3%) (25), electroconvulsive therapy (48.0%) (26) and standard FDA-approved repetitive transcranial magnetic stimulation (rTMS) protocols (5.9%) (27). However, Lan et al. found that iTBS (one sessions/day) and high-frequency rTMS appeared to be equally effective in alleviating depressive symptoms for patients with TRD (10). A recent meta-analysis of RCTs (*n* = 239) found that the study-defined response was greater for active accelerated iTBS (≥2 sessions of iTBS daily) than sham stimulation (13).

The short duration protocol (5 days) of SNT is a non-invasive brain stimulation with proven efficacy in TRD which could be used in emergency or inpatient settings where rapid-acting treatments are needed. As previously described (16, 17, 21), this protocol for SNT consisted of 5 consecutive days (90,000 total pulses) with ten iTBS sessions per day (18,000 pulses/day and a 50-min intersession interval per session) delivered to the region of the left dorsolateral prefrontal cortex (DLPFC). This protocol was designated SNT, to distinguish it from other accelerated iTBS protocols which do not have a high overall pulse dose of stimulation (SNT versus standard iTBS protocols: 90,000 versus 18,000 pulses) and individualized targeting using fcMRI (28, 29). This systematic review of studies with iTBS at high doses involved different intersession intervals per session. Therefore, one single-arm prospective study with its protocol for SNT consisting of 5 consecutive days (18,000 pulses/day, 90,000 total pulses and a 60-min intersession interval per session) was also included (24). However, the individual contribution of each element in the improvement of TRD outcomes is unclear, and this should be further examined.

As a rapid therapeutic intervention for TRD, SNT seems to be comparable to glutamatergic modulators like esketamine (the S-enantiomer of ketamine) (30), exhibiting a greater affinity for the N-methyl-d-aspartate receptor compared to the R-enantiomer (31). The administration of esketamine via intravenous (32) or intranasal (31) routes has a rapid onset of antidepressant effects. For example, Daly et al. found that esketamine administered intranasally at doses of 28, 56, and 84 mg appeared to be effective in treating TRD (31). A retrospective study found that accelerated high-frequency rTMS (four times daily for five consecutive days over the left DLPFC) appears to be more effective than intranasal esketamine (33). However, there are currently no head-to-head comparison studies on TMS and esketamine in treating TRD.

This systematic review has several limitations. First, only one RCT (17) was detected and the total sample size of the included studies (*n* = 63) was relatively small. Second, of the included four studies, three (16, 17, 21) were conducted by the same team at a single site, limiting generalizability of these findings. Third, the systematic review was not registered as this is not compulsory in most academic journals. Fourth, long-term follow up period (e.g., longer than 3 months) was not adopted in included studies, although the persistence of the antidepressant effect remains an important issue for TMS treatments, with several studies emphasizing the urgency of developing maintenance protocols to prevent potential relapses (34). Despite these limitations, this systematic review preliminarily found that SNT protocol appeared to be effective and well tolerated by patients with TRD. SNT is distinct from standard once daily TMS. An advantage of standard once daily TMS (treatment time 40 min) is that it allows time for supportive care to be provided by staff. Accelerated treatment offers considerable alternative advantages which will call for reorganization and reorientation of treatment centers. Future research is warranted to confirm and expand the utilization of SNT as an adjunctive treatment for TRD.

## Data availability statement

The original contributions presented in the study are included in the article/supplementary material, further inquiries can be directed to the corresponding authors.

## Author contributions

X-JL: Data curation, Writing – original draft. D-BC: Data curation, Writing – original draft. Q-ML: Data curation, Writing – original draft. Z-JQ: Writing – original draft. SP: Writing – review & editing. WZ: Writing – original draft. Y-TX: Writing – review & editing.
